# C-Reactive Protein/Albumin Ratio (CAR)-Integrated IMDC Model Improves Risk Stratification in Metastatic Renal Cell Carcinoma

**DOI:** 10.3390/cancers18091413

**Published:** 2026-04-29

**Authors:** Aaron I. Ahdoot, Cesare Saitta, Giuseppe Garofano, Dhruv Puri, Margaret F. Meagher, Benjamin H. Baker, Dattatraya Patil, Hajime Tanaka, Yosuke Yasuda, Masaki Kobayashi, William A. Langbo, Likhit Agrawal, Waleed Sabir, Shreya Kashyap, Sanjana Karamcheti, Srinivas Vourganti, Shohei Fukuda, Yasuhisa Fujii, Viraj Master, Edward Cherullo, Michael A. Liss, Giacomo Musso, Ithaar H. Derweesh

**Affiliations:** 1 Department of Urology, UC San Diego School of Medicine, San Diego, CA 92093, USAskaramcheti@health.ucsd.edu (S.K.); liss@health.ucsd.edu (M.A.L.); giacomo200197@gmail.com (G.M.); 2 Department of Urology, Emory University School of Medicine, Atlanta, GA 30322, USA; 3 Department of Urology, Tokyo Institute of Science, Tokyo 152-8550, Japany-fujii.uro@tmd.ac.jp (Y.F.); 4 Department of Urology, Rush University Medical Center, Chicago, IL 60612, USA; william_a_langbo@rush.edu (W.A.L.); waleed_sabir@rush.edu (W.S.);

**Keywords:** albumin, C-reactive protein, metastatic renal cell carcinoma, risk stratification, survival analysis

## Abstract

**Background/Objectives**: To evaluate the utility of the addition of C-reactive protein/albumin ratio (CAR) to the International Metastatic Renal Cell Carcinoma Database Consortium (IMDC). **Methods**: A retrospective multicenter analysis was conducted. The main outcomes were overall survival (OS) and all-cause mortality (ACM). Receiver operating characteristic (ROC) analysis determined the optimal CAR cutoff. Cox multivariable analysis (MVA) identified independent predictors for ACM. Kaplan–Meier analysis (KMA) evaluated OS stratified by IMDC risk groups. C-index assessed predictive accuracy of the CAR-augmented model. **Results**: We analyzed 392 patients (80 favorable-IMDC, 298 intermediate-IMDC, and 14 poor-risk). ROC revealed an optimal elevated CAR as ≥2.53. High CAR (HCAR) was an independent predictor of ACM on MVA (HR = 1.84, *p* < 0.001). KMA comparing IMDC favorable/intermediate/poor-risk revealed 3-year OS of 86.8%/57.9%/50.0%, *p* = 0.001. Subdivision by CAR revealed alignment between favorable-HCAR with intermediate–normal-CAR (NCAR) and intermediate-HCAR with poor-risk. KMA with the addition of CAR to the IMDC, demonstrating 3-year OS 91.0%, 72.7%, and 48.4% across favorable, intermediate, and poor-risk, respectively. Based on the alignment of favorable-HCAR with intermediate-NCAR, and intermediate-HCAR with poor-risk, a CAR-augmented IMDC algorithm was developed in which favorable- and intermediate-risk patients with NCAR are considered suitable CN candidates, while intermediate-HCAR and poor-risk patients are unlikely to benefit from surgery. The CAR augmented model showed improved predictive capability compared to the IMDC with a C-index of 0.70 vs. 0.67. **Conclusions**: Integrating CAR into the IMDC model enhances prognostic precision for CN selection in mRCC. This approach identifies intermediate-NCAR as appropriate CN candidates based on favorable survival, while sparing intermediate-HCAR from surgery.

## Simple Summary

In this study, we evaluate whether adding a simple blood-based marker, the C-reactive protein-to-albumin ratio, can improve how we predict outcomes in patients with metastatic renal cell carcinoma. Current risk models help guide treatment decisions, including whether patients are likely to benefit from cytoreductive nephrectomy, but they do not always capture important differences between patients. We aimed to determine whether incorporating this marker could better identify which patients are most likely to benefit from cytoreductive nephrectomy. Our findings show that including this measure improves the accuracy of existing risk models and more clearly separates patients with better or worse expected survival. This approach may support more personalized treatment decisions, helping avoid unnecessary surgery in patients unlikely to benefit while identifying those who may have improved outcomes. Overall, our work helps refine risk prediction and improve decision-making in metastatic renal cell carcinoma.

## 1. Introduction

Despite downward stage migration due to the increased diagnosis of small and asymptomatic renal masses, renal cell carcinoma (RCC) remains the deadliest common urologic malignancy, with up to 17% of patients presenting up front with metastases, and another 25% to 40% progressing to metastases despite surgical resection [1,2]. Since its introduction in 2009, the International Metastatic Renal Cell Carcinoma Database Consortium (IMDC) has served as the reference standard prognostic model for metastatic renal cell carcinoma (mRCC), integrating clinical and laboratory variables to stratify patient risk [3,4]. The C-reactive protein-to-albumin ratio (CAR)—a combination of C-reactive protein (CRP), an acute-phase inflammatory marker, and serum albumin, a marker of nutritional and functional status—is a simple, cost-effective indicator of poor outcomes in serious illness and several solid malignancies [5,6,7,8,9,10,11]. In localized RCC, elevated CAR has been independently associated with worse survival outcomes, and in mRCC, studies have demonstrated that an elevated CAR correlates with significantly shorter overall survival and progression-free survival, even among patients receiving targeted therapies [12,13,14,15,16,17,18,19,20]. Despite these promising associations, the integration of CAR into existing prognostic tools such as the IMDC model has not yet been systematically explored in patients receiving both surgical and systemic therapy.

In particular, the issue of patient selection for cytoreductive nephrectomy (CN) continues to challenge clinicians. While historical and real-world data support the use of CN in select clinical circumstances—demonstrating significant survival benefits in appropriately chosen patients—recent clinical trials such as CARMENA and SURTIME have questioned the broad, reflexive utilization of CN, showing that systemic therapy alone may be non-inferior to CN followed by systemic therapy, especially in patients with intermediate- or poor-risk features [21,22]. Current guidelines from the National Comprehensive Cancer Network and the American Society of Clinical Oncology recommend CN only for select patients, typically those with IMDC favorable- or intermediate-risk, good performance status, and limited metastatic burden [23,24]. Given these uncertainties, we sought to evaluate the utility of the CAR as a supplemental prognostic marker in mRCC patients stratified by IMDC criteria, aiming to further refine risk stratification and help guide personalized treatment decisions regarding CN.

## 2. Materials and Methods

### 2.1. Patient Population

We conducted a retrospective analysis utilizing the International Marker Consortium for Renal Cancer registry (INMARC). Centers contributing to the study included the University of California, San Diego; Emory University; Rush University; and Tokyo Institute of Science. Institutional review board approval was obtained at each center. Our protocols and procedures for evaluation, surgery, and follow-up have been previously described and were conducted according to the standard-of-care and current clinical guidelines [25,26,27,28,29,30]. Cross-sectional imaging of the chest, abdomen, and pelvis, along with preoperative laboratory testing, including CRP and albumin, was used to evaluate patients with suspected renal neoplasms. The overall study cohort included patients with metastatic RCC (mRCC) who either presented with metastatic disease at initial diagnosis (synchronous mRCC) or developed metastatic disease during follow-up after an earlier non-metastatic presentation (metachronous mRCC). All included patients ultimately underwent cytoreductive nephrectomy. For the development of the CAR-integrated IMDC clinical decision algorithm, analyses were restricted to patients with synchronous metastatic disease at diagnosis. All mRCC were histologically confirmed by biopsy or resection of the primary and/or metastatic site. Patients were managed in an interdisciplinary manner regarding recommendations to perform cytoreductive surgery and/or metastatectomy.

We excluded patients with localized RCC, patients who had benign cortical neoplasms, patients with upper tract urothelial neoplasms, and patients with mRCC who did not have a preoperative CAR, absolute neutrophil count (ANC), corrected calcium, platelet count, hemoglobin, and Karnofsky performance score (KPS).

### 2.2. Data Collection

Demographic data included age at diagnosis, sex, race, and body mass index (BMI). Disease characteristics included primary tumor size and location/number of metastases, and preoperative platelet count, absolute neutrophil count (ANC), CAR, hemoglobin, corrected calcium, KPS, time to systemic therapy, and survival outcomes (overall survival (OS)/all-cause mortality (ACM)), which were calculated from the date of metastatic RCC diagnosis to the date of last follow-up or death. For patients with synchronous metastatic disease, this corresponded to the initial diagnosis date; for those who developed metastatic disease during follow-up, this corresponded to the date metastatic disease was first identified. Patients were included regardless of whether they received systemic therapy before or after cytoreductive nephrectomy; in all cases, preoperative values were recorded at the clinical evaluation immediately preceding the surgical procedure.

### 2.3. Statistical Analysis

The main outcome was (OS)/(ACM). Descriptive analysis was performed by stratifying patients into their respective IMDC strata. Group comparison was conducted using Pearson chi-square for categorical variables and Kruskal–Wallis for continuous variables. Receiver operating characteristic (ROC) analysis was performed to identify the cohort-specific CAR threshold associated with the greatest discriminatory ability for survival outcomes in the present study population. Cox proportional hazard multivariable analysis (MVA) was conducted to elucidate independent preoperative predictors for ACM, including elevated CAR, platelet count, ANC, hemoglobin, corrected calcium, follow-up time, time to systemic therapy, sex, age at diagnosis, and BMI. The dataset was first divided into training and testing sets with a 70:30 split. Primary analyses were based on conventional survival methods, including ROC analysis, Cox proportional hazards modeling, Kaplan–Meier analysis, and Harrell’s C-index. Additional machine-learning survival approaches, including Random Survival Forest (RSF), Survival Support Vector Machine (Survival SVM), and Gradient Boosting (XGBoost), were explored for internal supportive model development, but were not used as the primary basis for the study’s clinical conclusions. Kaplan–Meier analysis (KMA) was performed to evaluate overall survival stratified by IMDC risk category and further subdivided by normal versus elevated preoperative CAR.

Primary prognostic analyses were performed in the overall cohort of mRCC patients undergoing cytoreductive nephrectomy. For algorithm development, analyses were restricted to the subcohort of patients with synchronous mRCC, excluding those who developed metastatic disease during follow-up, because the clinical question of upfront cytoreductive nephrectomy selection is most relevant in this population. Differences between survival curves were assessed using the log-rank test. Predictive performance for overall survival was compared between the standard IMDC model and the CAR-augmented model using Harrell’s C-index. Based on survival patterns observed in the synchronous mRCC cohort, a combined CAR–IMDC decision algorithm was derived to inform the selection of patients most likely to benefit from cytoreductive nephrectomy.

The dataset was analyzed using SPSS 29.0.2.0 (IBM, Chicago, IL, USA) and Python version 3.13.5 (Python Software Foundation, Wilmington, DE, USA). All tests were 2-sided with an alpha level of 0.05.

## 3. Results

**Figure 1:**
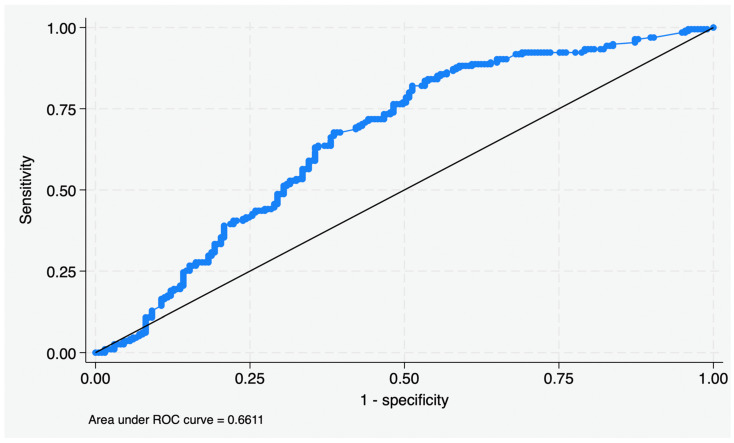
ROC curve for optimal CAR threshold of ≥2.53.

A total of 392 patients met the inclusion criteria (median follow-up of 30.4 months), which were stratified into 80 favorable-, 298 intermediate-, and 14 poor-risk (Table 1). ROC analysis revealed an AUC of 0.66 and identified a CAR threshold of ≥2.53 as the optimal cutoff for dichotomized analyses within the present cohort (Figure 1). Given the modest discriminatory performance, this threshold should be interpreted as a cohort-derived prognostic cutoff rather than a universally applicable clinical standard. Most patients (52.8%) had an elevated preoperative CRP, comprising 60.2% of the intermediate-risk group and 85.7% of the poor-risk group, though the difference remained significant (*p* < 0.001) across the three cohorts.

There were significant differences in the median age (*p* = 0.005), race (*p* < 0.001), sex (*p* < 0.001), and median BMI (*p* < 0.001) between the three IMDC groups. The median primary tumor size differed significantly across the three risk groups: 5.0 cm in the favorable-risk group, 9.0 cm in the intermediate-risk group, and 9.5 cm in the poor-risk group (*p* < 0.001). There were also significant differences in five out of the six IMDC criteria between the strata, including KPS below 80% (*p* < 0.001), preoperative hemoglobin below the normal limit (*p* < 0.001), and preoperative ANC, platelet count, and corrected hypercalcemia (*p* < 0.001), with patients in the poor-risk group having the largest proportion of patients meeting the criteria.

Table 2 demonstrates MVA for survival outcomes. MVA revealed that elevated preoperative CAR was an independent risk factor associated with worsened ACM (HR = 1.84, *p* < 0.001). In addition, other variables significantly associated with ACM were elevated ANC (HR = 1.97, *p* = 0.003) and elevated corrected calcium (HR = 2.19, *p* = 0.003). Remaining IMDC predictors, namely anemia (HR = 0.70, *p* = 0.12), thrombocytosis (HR = 0.87, *p* = 0.63), time to systemic therapy (HR = 0.67, *p* = 0.41), and KPS (HR = 1.48, *p* = 0.52), did not show an independent association. Using clinically interpretable survival modeling, the Cox-based CAR-augmented model demonstrated improved predictive performance compared with the standard IMDC model, with a C-index of 0.70 versus 0.67. Exploratory machine-learning approaches were evaluated during internal model development but were not used as the basis for the primary reported conclusions.

**Figure 2:**
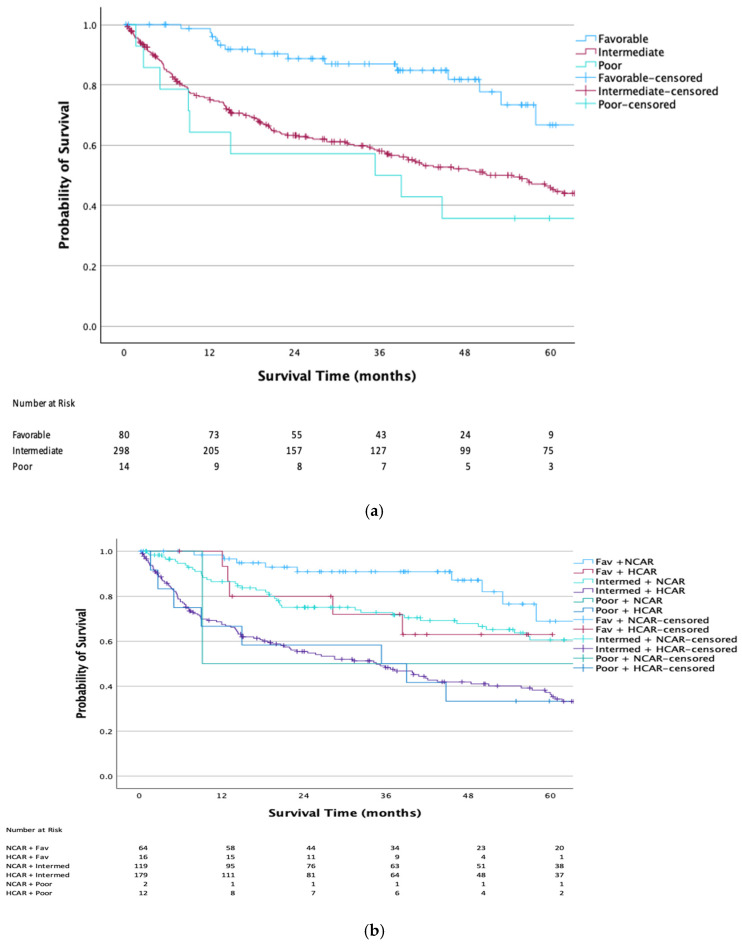

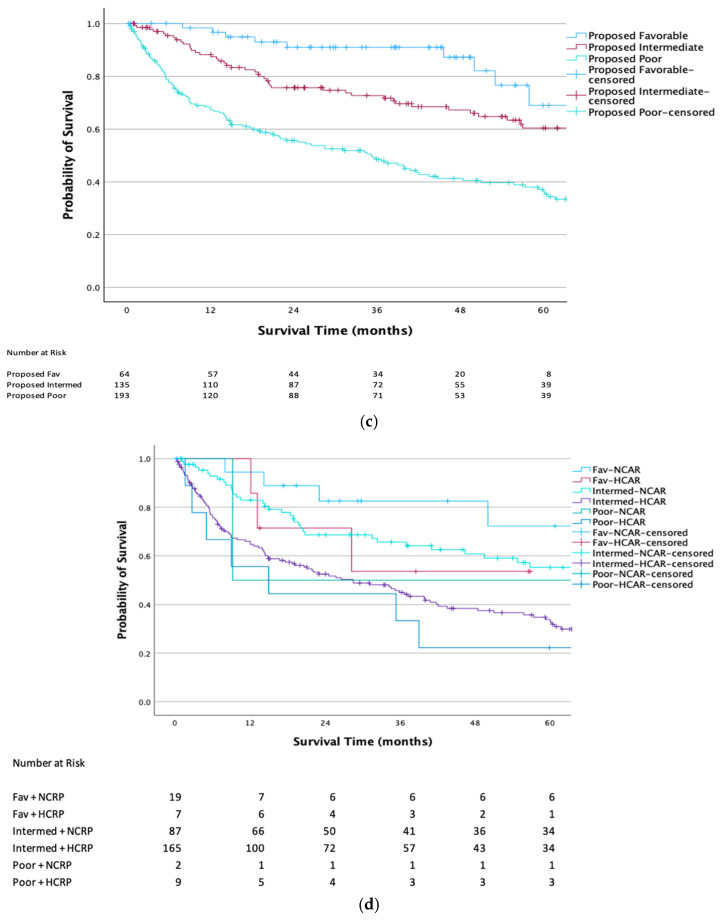
KMA for OS using (**a**) IMDC Strata, (**b**) subdivision of IMDC Strata based on normal or high CAR, (**c**) proposed IMDC Strata, and (**d**) subdivision of IMDC Strata of patients presenting with metastasis with normal or high CAR. Legend: Fav = Favorable-risk; Intermed = Intermediate-risk; Poor = Poor-risk; NCAR = normal CAR; and HCAR = High CAR.

Figure 2 demonstrates that KMA comparing IMDC favorable-, intermediate-, and poor-risk groups revealed 3-year overall survival (OS) rates of 86.8%, 57.9%, and 50.0%, respectively (log-rank *p* < 0.001; Figure 2a). To further explore prognostic discrimination, IMDC strata were subdivided by normal CAR (NCAR) and high CAR (HCAR). Among favorable-risk patients, OS was 91.0% for those with NCAR (median OS 81.1 months) versus 72.0% for those with HCAR (median OS not reached). In the intermediate-risk group, 3-year OS was 72.8% for NCAR and 48.3% for HCAR (median OS 74.0 months and 34.6 months, respectively). Among poor-risk patients, 3-year OS was 50.0% in both CAR subgroups (median OS 9.2 months for NCAR and 35.3 months for HCAR; Figure 2b). Given the observed similarities in 3-year OS within certain CAR-stratified subgroups, we proposed a revised risk model. The new stratification grouped patients into: (1) new favorable (current favorable with NCAR); (2) new intermediate (current favorable with HCAR and current intermediate with NCAR); and (3) new poor-risk (current intermediate with elevated CAR and all poor-risk patients). This model yielded 3-year OS rates of 91.0%, 72.7%, and 48.4% (median OS of 77.0, 73.6, and 35.0 months), respectively (Figure 2c), and maintained a statistically significant difference in OS (*p* < 0.001).

Because the decision regarding upfront cytoreductive nephrectomy is most relevant in patients with synchronous metastatic disease, we next performed a subgroup analysis limited to patients presenting with metastasis at diagnosis. To derive a clinically applicable decision algorithm, we restricted the analysis to patients presenting with metastatic disease at diagnosis and excluded those who developed metastatic disease during follow-up. Within this metastatic-at-presentation cohort (*n* = 289), patients were distributed across six CAR–IMDC subgroups: favorable-risk with normal CAR (NCAR; *n* = 19), favorable-risk with high CAR (HCAR; *n* = 7), intermediate-risk with NCAR (*n* = 87), intermediate-risk with HCAR (*n* = 165), poor-risk with NCAR (*n* = 2), and poor-risk with HCAR (*n* = 9; Figure 2d).

KMA of overall survival demonstrated alignment in outcomes between favorable- and intermediate-risk patients with NCAR, as well as between intermediate-risk patients with HCAR and those classified as poor-risk, supporting regrouping based on combined IMDC and CAR stratification. Favorable- and intermediate-risk patients with NCAR exhibited prolonged survival, with a median overall survival of 74.0 months (IQR 18.9–111.5), whereas intermediate-risk patients with HCAR and poor-risk patients experienced substantially worse outcomes, with a median overall survival of 28.5 months (IQR 6.3–67.9).

**Figure 3:**
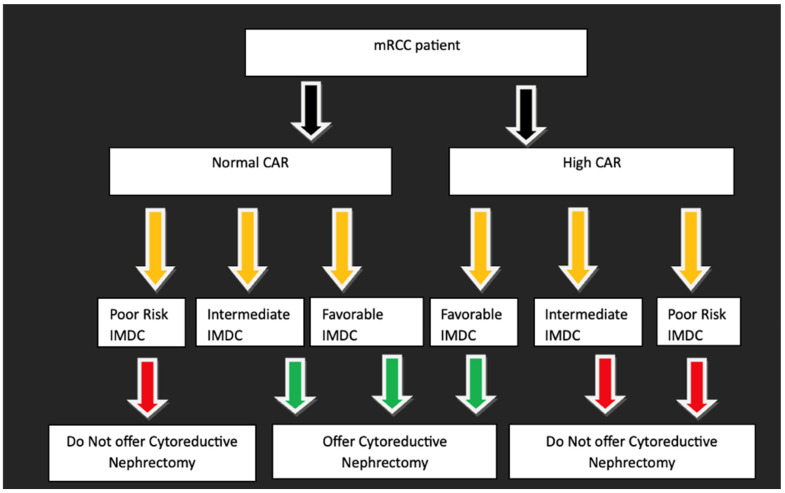
Proposed clinical reasoning algorithm for cytoreductive nephrectomy selection.

Based on these survival patterns, we proposed a CAR-augmented IMDC algorithm for patients presenting with metastatic disease, in which favorable- and intermediate-risk patients with NCAR are considered appropriate candidates for cytoreductive nephrectomy, while intermediate-risk patients with HCAR and poor-risk patients are unlikely to derive meaningful benefit from surgery (Figure 3). Application of this algorithm stratified 113 patients (39.1%) into a favorable surgical group, comprising favorable-risk IMDC and intermediate-risk NCAR, and 176 patients (60.9%) into an unfavorable surgical group, comprising poor-risk IMDC and intermediate-risk HCAR. Although all patients in the study cohort underwent cytoreductive nephrectomy, the unfavorable group accounted for the majority of death events (125 of 175 deaths, 71.4%) with a median OS of 28.5 months (IQR 6.3–67.9) compared to a median OS of 74.0 months (IQR 18.9–111.5) in the favorable group. Application of the proposed algorithm suggests that these 176 intermediate-HCAR and poor-risk patients could have avoided upfront cytoreductive nephrectomy.

## 4. Discussion

In this multicenter retrospective study, we examined the utility of adding CAR to the existing IMDC model, considering that CAR is a known predictor of outcomes in localized and metastatic RCC [12,13,14,15,16,17,18,19,20]. We found that CAR was a significant, independent predictor of OS in patients with mRCC who underwent cytoreductive partial or radical nephrectomy. Elevated CAR was correlated with a significantly increased risk of ACM, with a stronger association than all current IMDC criteria. Incorporation of CAR into the IMDC as an independent predictor led to a significant improvement in the model’s ability to predict OS. We also found that elevated CAR could be used to further stratify patients within the IMDC intermediate-risk group to more accurately predict their probability of OS. While we internally validated our analyses, our findings nonetheless call for external validation and further investigation into the integration of CAR in an updated IMDC model to more accurately predict outcomes in mRCC and to guide clinical decision-making. Importantly, the conclusions of this study are based on conventional, clinically interpretable survival analyses, whereas machine-learning approaches were explored only in a supportive manner during internal model development.

Emerging analyses suggest that CAR is associated with survival outcomes in mRCC. Konishi et al., in a retrospective analysis of 176 patients with mRCC, noted that elevated CAR was predictive of worsened ACM (HR = 1.72, *p* = 0.001) [18]. Furthermore, Barua et al., in a retrospective study of 31 patients with mRCC, also noted that CAR correlated with worsened ACM (HR = 4.445, *p* = 0.001) [19]. These findings are similar to our analysis, which noted that elevated CAR (HR = 1.84, *p* < 0.001) was a predictor for ACM, and this association was stronger than that of any other IMDC criterion. Taken together, these findings support the prognostic relevance of CAR in mRCC and underscore its potential utility as a complementary marker in existing risk stratification models.

Building on this evidence for CAR’s individual prognostic value, recent efforts have explored its integration into established risk models—most notably the IMDC—to enhance their predictive performance in mRCC. Heng et al. externally validated the IMDC model in a large, multicenter cohort of 1028 patients from 13 international institutions, reporting a C-index of 0.71 for predicting overall survival [31]. This performance likely reflects the benefits of a large sample size and institutional diversity in enhancing model reliability. Since then, several studies have suggested that CAR can improve the prognostic accuracy of OS in metastatic RCC. Proctor et al. and Brown et al. found that a modified Glasgow Prognostic Score, a powerful prognostic factor that considers both CRP and albumin in cancer outcomes, exhibited improved prediction of OS compared to the IMDC in mRCC patients on immunotherapy [32,33]. Tamura et al. showed that a novel model incorporating CRP, albumin, and neutrophil-to-lymphocyte ratio in patients receiving first-line targeted therapy improved the C-index from 0.67 to 0.72 [34]. Moreover, Konishi et al. demonstrated that in patients treated exclusively with TKIs, the addition of CAR improved the AUC of the IMDC from 0.689 to 0.720 [19].

However, these prior studies often focused on narrowly defined treatment groups or were limited to specific therapy classes, reducing their generalizability to the broader mRCC population. In contrast, our study evaluated a contemporary, diverse cohort in which every patient underwent surgical resection and a vast majority received systemic therapy spanning the full range of modern options, including TKIs, immune checkpoint inhibitors, and combination regimens, enhancing the generalizability of our findings. While our cohort yielded a C-index of 0.67 for the traditional IMDC, our CAR-augmented model demonstrated superior predictive accuracy with a C-index of 0.70, underscoring the model’s robustness and potential clinical utility. Although the improvement in C-index from 0.67 to 0.70 was modest, even incremental gains in discrimination may be relevant when added to an established prognostic model such as IMDC, particularly if they improve risk refinement within clinically heterogeneous groups such as intermediate-risk patients. We anticipate that applying this CAR-augmented model to larger multicenter datasets could further amplify its prognostic value, supporting its integration into future risk stratification frameworks for mRCC.

Our analyses add to the emerging literature about augmenting and improving the IMDC by utilizing a composite index of two independent variables and utilizing a unique subpopulation of mRCC patients, namely those undergoing CN. While the IMDC has served as an integral tool for mRCC patients, it is limited by its tendency to group most patients in a heterogeneous intermediate-risk cohort [35]. Our study also aligns with the prior literature examining the utility of CAR in improving IMDC risk-stratification. Konishi et al. and Uzun et al. demonstrated that baseline CAR levels could subdivide intermediate-risk mRCC patients into distinct prognostic groups with a significant difference in OS [18,36]. Similarly, our study illustrated CAR’s utility in stratifying survival outcomes within IMDC groups. While the traditional IMDC model stratified patients into favorable-, intermediate-, and poor-risk groups with 3-year OS rates of 86.8%, 57.9%, and 50.0% (*p* < 0.001), CAR further distinguished outcomes within these categories. In favorable-risk patients, 3-year OS was 91.0% with normal CAR vs. 72.0% with elevated CAR; in the intermediate group, it was 72.8% vs. 48.3%, respectively. Poor-risk patients showed no CAR-based difference (50.0% OS). Notably, the CAR threshold identified in our study was substantially higher than those reported in prior mRCC series, including Konishi et al. and Uzun et al., likely reflecting differences in cohort composition, treatment setting, and analytic approach. Because our cohort consisted exclusively of patients undergoing cytoreductive nephrectomy in a multicenter contemporary setting, the cutoff identified here should be interpreted as a cohort-derived prognostic threshold rather than a universally applicable clinical standard.

Specifically, our CAR-augmented IMDC algorithm better discriminates survival outcomes within intermediate-risk patients, identifying those with high CAR as having significantly worse overall survival compared to those with low CAR, a distinction not achieved by IMDC risk factors alone [19,36]. In contrast, the CARMENA trial questioned the broad use of CN, showing that systemic therapy alone was non-inferior to CN plus systemic therapy, especially in patients with intermediate- or poor-risk features [21]. The CAR-augmented IMDC algorithm addresses a key limitation of CARMENA by refining risk stratification within the intermediate-risk group, enabling more precise identification of patients likely to benefit from CN.

Importantly, while the overall cohort included both patients with synchronous metastatic disease and those who developed metastases during follow-up, development of the CAR-augmented IMDC algorithm was restricted to patients presenting with metastatic disease at diagnosis, as the question of upfront cytoreductive nephrectomy selection is most relevant in this setting. In our study, alignment of overall survival across CAR-IMDC risk strata—specifically, favorable- and intermediate-risk patients with normal CAR aligning with one another, and intermediate-risk patients with high CAR aligning with poor-risk patients—enabled the development of a CAR-augmented IMDC algorithm that more precisely stratifies candidates for cytoreductive nephrectomy. Within this framework, favorable- and intermediate-risk patients with normal CAR are considered appropriate surgical candidates, whereas intermediate-risk patients with high CAR and those classified as poor-risk are unlikely to derive meaningful benefit from nephrectomy. A key implication of this algorithm is the identification of patients in whom cytoreductive nephrectomy may confer limited survival benefit despite being performed in contemporary practice. While our cohort only included surgical patients, the median OS of 28.5 months for the unfavorable group is remarkably consistent with the 25.2–31.2 months reported for the non-surgical arm of the CARMENA trial. This suggests that for patients with high inflammatory burden, the survival outcome with surgery is comparable to historical expectations for systemic therapy alone. These findings suggest that a substantial proportion of patients—particularly those with high inflammatory burden or poor-risk disease—may continue to undergo cytoreductive nephrectomy without a clear survival advantage.

In contrast, intermediate-risk patients with normal CAR who underwent cytoreductive nephrectomy demonstrated substantially improved outcomes, with a median overall survival of 74.0 months. Together, these findings support a selective role for cytoreductive nephrectomy and highlight the potential of this algorithm to refine patient selection by identifying individuals unlikely to benefit from surgery while preserving surgical intervention for those with favorable biology. Accordingly, this proposed CAR-augmented framework should be interpreted as a conceptual, hypothesis-generating model to support future study, rather than as a validated decision pathway for independently offering or withholding cytoreductive nephrectomy.

Our study is not without limitations. Given the retrospective study design, there is an inherent risk of bias; specifically, the exclusion of patients with incomplete preoperative laboratory or clinical data required for CAR and IMDC classification may have introduced selection bias. Additionally, we did not account for potential infectious and inflammatory processes that can elevate CRP and potentially confound the analysis. Nevertheless, CRP and albumin were obtained preoperatively in patients without ongoing acute infections. The lack of standardized systemic therapy protocols and variability in follow-up further limit the generalizability of our findings. Furthermore, the ROC-derived CAR cutoff demonstrated only modest discriminatory performance, and CAR was analyzed as a dichotomized rather than continuous variable. Future studies should evaluate CAR as a continuous predictor and externally validate whether the cohort-derived threshold identified here is reproducible across broader mRCC populations. Moreover, all patients included underwent cytoreductive nephrectomy, so the results may not be applicable to patients who did not receive surgical cytoreduction. In addition, as all patients in this cohort underwent CN, we cannot definitively conclude that the unfavorable group would have fared equally well without surgery; however, the alignment of their survival with historical non-surgical data is a significant finding. External validation is necessary to confirm the utility of adding CAR to the IMDC model.

## 5. Conclusions

Elevated CAR is a strong, independent predictor of overall survival, outperforming several traditional IMDC risk factors. Incorporation of CAR into the IMDC framework improved prognostic discrimination and enabled a refined risk stratification algorithm that more precisely identifies candidates for cytoreductive nephrectomy. Within this model, intermediate-risk patients with normal CAR demonstrated favorable survival outcomes comparable to favorable-risk patients, supporting their consideration for CN, whereas intermediate-risk patients with high CAR and poor-risk patients derived limited survival benefit and may be better managed with systemic therapy alone. By distinguishing patients likely to benefit from CN from those in whom surgery may be futile, this approach offers a more granular and clinically actionable framework for treatment selection. Prospective validation is warranted to confirm these findings.

## Figures and Tables

**Table 1:** Demographics and clinical disease characteristics.

Variable	Total Cohort (*n* = 392)	Favorable Risk (*n* = 80)	Intermediate Risk (*n* = 298)	Poor Risk (*n* = 14)	*p* Value
Median Age (years, IQR)	63.0 (14.7)	62.0 (15.0)	64.0 (15.0)	59.0 (18.3)	0.005
Sex (*n*, %)					<0.001
Female	231 (58.9%)	31 (38.8%)	191 (64.1%)	9 (64.2%)	
Male	161 (41.1%)	49 (61.2%)	107 (35.9%)	5 (35.7%)	
Race (*n*, %)					<0.001
Caucasian	238 (60.7%)	39 (48.8%)	192 (48.2%)	7 (50.0%)	
Black	23 (5.9%)	4 (5.0%)	18 (6.0%)	1 (7.1%)	
Asian	64 (16.3%)	4 (5.0%)	56 (18.8%)	4 (28.6%)	
Latino	57 (14.5%)	31 (38.8%)	24 (8.1%)	2 (14.3%)	
Other	10 (2.6%)	2 (2.5%)	8 (2.7%)	0	
Median BMI (kg/m^2^, IQR)	27.5 (7.2)	29.2 (8.2)	27.2 (7.0)	24.5 (6.1)	<0.001
Karnofsky Performance Score < 80% (*n*, %)	6 (1.5%)	0	4 (1.3%)	2 (14.3%)	<0.001
Median Primary Tumor Size (cm, IQR)	8.0 (6.4)	5.0 (4.2)	9.0 (6.5)	9.5 (5.5)	<0.001
Time to Systemic Therapy > 1 year (*n*, %)	18 (4.6%)	0	17 (5.7%)	1 (7.1%)	0.086
Hemoglobin < Lower Limit of Normal (*n*, %)	72 (17.3%)	0	63 (21.1%)	9 (64.3%)	<0.001
Absolute Neutrophil Count > Upper Limit of Normal (*n*, %)	252 (64.3%)	0	239 (80.2%)	13 (92.9%)	<0.001
Platelet Count > Upper Limit of Normal (*n*, %)	31 (7.9%)	0	19 (6.4%)	12 (85.7%)	<0.001
Corrected calcium > Upper Limit of Normal (*n*, %)	24 (6.1%)	0	15 (5.0%)	9 (64.3%)	<0.001
Surgery Type					<0.001
Partial Nephrectomy	77 (19.6%)	41 (51.3%)	36 (12.1%)	0	
Radical Nephrectomy	315 (80.4%)	39 (48.7%)	262 (87.9%)	14 (100.0%)	
Neoadjuvant Systemic Therapy (*n*, %)	77 (19.6%)	21 (26.3%)	53 (17.8%)	3 (21.4%)	0.235
Medical Treatment Type (*n*, %)					<0.001
Chemotherapy	7 (1.8%)	3 (3.8%)	2 (0.6%)	2 (14.2%)	
Immunotherapy	127 (32.4%)	39 (48.8%)	88 (29.5%)	0 (0%)	
Chemotherapy & Immunotherapy	10 (2.6%)	5 (6.3%)	4 (1.3%)	1 (7.1%)	
Tyrosine Kinase Inhibitor	161 (41.1%)	22 (27.5%)	133 (44.6%)	6 (42.9%)	
Immunotherapy & Tyrosine Kinase Inhibitor	21 (5.4%)	5 (6.3%)	16 (5.4%)	0	
None	11 (2.8%)	3 (3.8%)	7 (2.3%)	1 (7.1%)	
Elevated CAR (*n*, %)	207 (52.8%)	16 (25.8%)	179 (60.1%)	12(85.7%)	<0.001
All-Cause Mortality (*n*, %)	195 (49.7%)	17 (21.3%)	168 (56.4%)	10 (71.4%)	<0.001
Median Follow-up (months, IQR)	30.4 (47.1)	38.5 (31.3)	25.7 (52.7)	37.2 (58.2)	0.282

**Table 2:** Multivariable Cox regression for ACM.

Variable	HR	95% CI	*p* Value
Increasing age (continuous)	1.00	0.99–1.02	0.38
Increasing BMI (continuous)	0.99	0.96–1.02	0.45
Male Sex (yes or no)	1.33	0.95–1.88	0.10
Karnofsky PS < 80% (yes or no)	1.48	0.45–5.611	0.366
Time to Systemic Therapy > 1 year (yes or no)	0.660	0.254–4.83	0.52
Hemoglobin < Lower Limit of NL (yes or no)	0.70	0.44–1.10	0.12
Platelets > Upper Limit of Normal (yes or no)	0.87	0.50–1.53	0.63
Neutrophils > Upper Limit of Normal (yes or no)	1.97	1.26–3.08	0.003
Corrected Calcium > Upper Limit Normal (yes or no)	2.19	1.30–3.70	0.003
CAR ≥ 2.53 (yes or no)	1.84	1.30–2.61	<0.001

## Data Availability

The data supporting the findings of this study are not publicly available due to privacy and ethical restrictions related to the use of patient-level clinical data from multiple institutions.

## Abbreviations

The following abbreviations are used in this manuscript:

ACM	All-cause mortality
ANC	Absolute neutrophil count
AUC	Area under the curve
BMI	Body mass index
CAR	C-reactive protein-to-albumin ratio
CRP	C-reactive protein
CN	Cytoreductive nephrectomy
C-Index	Concordance index
IMDC	International Metastatic Renal Cell Carcinoma Database Consortium
INMARC	International Marker Consortium for Renal Cancer
KMA	Kaplan–Meier analysis
KPS	Karnofsky performance score
MVA	Multivariable analysis
mRCC	Metastatic renal cell carcinoma
NCAR	Normal C-reactive protein-to-albumin ratio
HCAR	High C-reactive protein-to-albumin ratio
OS	Overall survival
RCC	Renal cell carcinoma
ROC	Receiver operating characteristic
RSF	Random survival forest
SVM	Support vector machine
TKI	Tyrosine kinase inhibitor
